# Skin tone corrected pulse oximetry models evaluated through reflective pulsatile Monte Carlo simulations

**DOI:** 10.1117/1.BIOS.2.3.032507

**Published:** 2025-08-13

**Authors:** Yan Tung Nicholas Chan, Megh Rathod, Daniel Franklin

**Affiliations:** a University of Toronto, Faculty of Applied Science and Engineering, Division of Engineering Science, Toronto, Ontario, Canada; b Translational Biology and Engineering Program, Ted Rogers Center for Heart Research, Toronto, Ontario, Canada; c University of Toronto, Faculty of Applied Science and Engineering, Institute of Biomedical Engineering, Toronto, Ontario, Canada

**Keywords:** pulse oximetry, skin tone bias, Monte Carlo simulation, arterial blood oxygen saturation correction models, equitable patient care

## Abstract

**Significance:**

Skin tone bias in pulse oximetry is frequently reported to lead to overestimation of arterial blood oxygen saturation (${\mathrm{SpO}}_{2}$) in individuals with darker skin tones. We introduce clinically applicable, skin tone corrected pulse oximetry models to address this disparity and promote more equitable patient care.

**Aim:**

We aim to mitigate skin tone bias in pulse oximetry by integrating a melanin correction factor into the conventional ratio-of-ratios approach. We employ pulsatile Monte Carlo simulation to systematically map the “ratio of ratios” (R) to ${\mathrm{SaO}}_{2}$ across varying melanin concentrations, thereby enabling comprehensive quantitative assessments of different ${\mathrm{SpO}}_{2}$ correction models.

**Approach:**

A six-layered skin model is implemented to approximate the microstructure of human skin, allowing diffuse reflectance computations through Monte Carlo methods. Pulsatile effects are captured by modeling diastolic and systolic phrases, facilitating the calculation of R values under diverse ${\mathrm{SaO}}_{2}$ and melanin conditions.

**Results:**

Skin tone induced error is quantified and mitigated by incorporating a melanin correction factor into conventional ratio-of-ratios. The inclusion of a melanin-dependent variable results in a substantial reduction in skin tone bias.

**Conclusions:**

Monte Carlo simulations offer precise control over both melanin content and ${\mathrm{SpO}}_{2}$, conditions that are challenging to replicate *in vivo*, and enable the development of robust ${\mathrm{SpO}}_{2}$ correction models. These findings represent a valuable step toward more equitable pulse oximetry practices, ultimately improving patient care across diverse skin tones.

Statement of Discovery This work utilizes Monte Carlo simulations to quantify skin tone induced error in pulse oximetry and proposes clinically applicable, skin tone corrected pulse oximetry models to address this disparity.

## 1 Introduction

Pulse oximetry enables the non-invasive estimation of arterial blood saturation and is a vital supplement to invasive blood gas analysis within critical care settings. The proliferation of inexpensive pulse oximeters also enables continuous blood saturation monitoring in diverse healthcare settings including surgery, pulmonary rehabilitation, obstructive sleep apnea screening, and remote patient monitoring with wearable devices.^1^^–^^4^ Despite the pivotal role of pulse oximeters in clinical decision-making, findings indicate that standard pulse oximeters frequently overestimate arterial blood oxygen saturation (${\mathrm{SpO}}_{2}$) (by 2% to 4%) in patients with darker skin tones.^5^^–^^8^ With ${\mathrm{SpO}}_{2}$ thresholds guiding interventions for hypoxia,^9^^,^^10^ even modest inaccuracies can have significant clinical repercussions, including higher rates of organ dysfunction and mortality.^11^ This bias hampers the timely detection of hypoxemia and disproportionately affects minority populations with darker skin pigmentation, as evidenced by increased rates of occult hypoxemia, reduced administration of supplemental oxygen, and delayed COVID-19 diagnosis and treatment.^8^^,^^12^^–^^14^ Skin tone dependent inaccuracies in pulse oximetry both contribute to and exacerbate existing inequalities in healthcare delivery.

Conventional pulse oximetry is performed through photoplethysmography (PPG) at two wavelengths, red (655 nm) and near-infrared (940 nm), and calculated through the ratio-of-ratios method to determine peripheral oxygen saturation (${\mathrm{SpO}}_{2}$).^15^ The ratio-of-ratios method is derived from a modified Beer–Lambert law and makes several assumptions that are hypothesized to contribute to skin tone bias in pulse oximeters—namely, that the optical path, length, and loss due to scattering are the same throughout the cardiac cycle and the same for red and infrared light across individuals.^16^ These oversimplifications neglect the contributions of non-pulsatile chromophores, most notably melanin, despite its differing absorption and scattering for red and near-infrared light.^17^

Several prior works have attempted to address skin tone bias through corrected ratio-of-ratios models,^18^^–^^25^ though no clinically viable solution has yet been established. For example, a recently published study employed individual typology angle (ITA) to adjust ${\mathrm{SpO}}_{2}$ measurements based on skin tone;^24^ although this approach reduces bias for individuals with darker skin tones, it increases errors for other skin tones. Similarly, a study using the Von Luschan Chromatic Scale (VLS) demonstrated improved hemoglobin estimates compared with commercial pulse oximeters yet provided no conclusive evidence that the proposed model effectively mitigated skin tone bias.^25^ Given the widespread reliance on ${\mathrm{SpO}}_{2}$ for critical medical decisions,^26^ there is an urgent need for a systematic evaluation of corrective ratio-of-ratios models to ensure equitable and accurate patient care.

Here, we use Monte Carlo simulations to empirically assess the impact of melanin on reflective pulse oximeters and evaluate corrective ratio-of-ratios models. We simulate light–tissue interactions throughout a series of six horizontally stacked skin layers, each characterized by specific structural and optical properties, including varying volume fractions of melanin, blood, water, and fat. Diffuse reflectance is computed by placing a source and detector on the tissue surface, approximating a reflectance pulse oximetry probe. The skin model is validated by fine-tuning its parameters until the simulated reflectance spectra agree closely with experimentally measured human finger data for both light and dark skin tones. Once validated, the model is extended to incorporate pulsatile dynamics, with diastolic and systolic simulations capturing changes in arterial blood volume and layer thickness. We extract the pulsatile (AC) component of a PPG signal from the diastolic and systolic diffuse reflectance, which is subsequently normalized by the baseline (DC) value. This AC/DC ratio forms the basis of the ratio-of-ratios (R). By systematically varying melanin volume fraction and arterial blood oxygen saturation (${\mathrm{SaO}}_{2}$), we map R to ${\mathrm{SaO}}_{2}$ under a broad range of conditions, thereby quantifying the extent of skin tone bias in pulse oximetry. Lastly, we introduce melanin correction factors into the ratio-of-ratios, thereby enabling multi-parameter curve-fitting that spans all relevant melanin values. By minimizing the fitting loss across this ${\mathrm{SaO}}_{2}$–R dataset, the skin tone induced error is substantially reduced. We propose multiple candidate models and select those that demonstrate the lowest overall bias. These candidate expressions have the potential to be integrated into clinical pulse oximetry systems to provide more equitable ${\mathrm{SpO}}_{2}$ measurements for individuals of all skin tones.

## 2 Methods

### 2.1 Monte Carlo Simulation of Light–Tissue Interactions

**Fig. 1:**
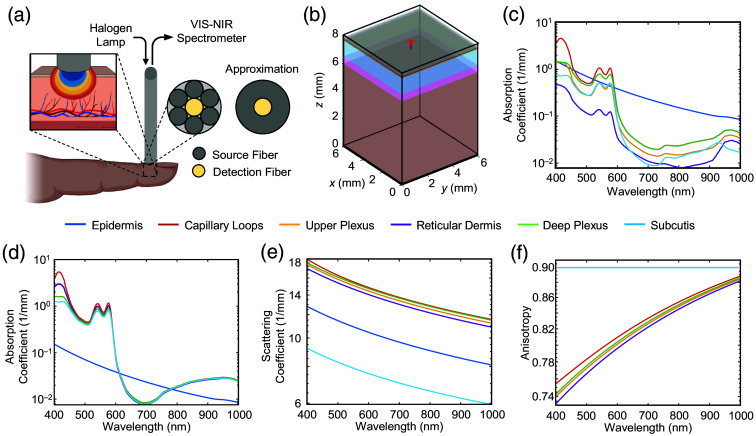
Simulation configuration and overview of optical parameters. (a) Reflectance probe configuration for measuring diffuse reflectance and approximation of source–detector geometry. (b) Simulation environment modeling microstructure composition of the human skin. Detailed chromophore volume fractions and structural properties, including layer thickness, are provided in Table 1. (c) Absorption coefficients of individual skin layers during diastole. (d) Absorption coefficient during systole, reflecting changes due to variations in blood volume and layer thickness over the cardiac cycle. The epidermal absorption spectrum remains constant, as it is the only layer that does not contain blood. (e) Scattering coefficient of the skin layers, incorporating both Rayleigh and Mie scattering. (f) Anisotropy values for each layer, specifying photon scattering angles during simulations. Both the scattering and anisotropy spectra are identical under diastolic and systolic conditions.

Monte Carlo simulation is a widely utilized technique for modeling the complex processes governing photon propagation within heterogeneous media, such as human skin.^27^ It is frequently employed to investigate the influence of skin tone in pulse oximetry.^28^^–^^31^ In this study, we used MCXLAB, an open-source light transport simulator in MATLAB,^32^ to conduct Monte Carlo simulations on an NVIDIA RTX 4090 GPU with $10^{8}\;\text{photons}$ per simulation (see Fig. S1 in the Supplementary Material for justification of photon count). Each simulation requires ∼1  min to complete—of which there are then 601 simulations over wavelength for diffuse reflectance. Pulsation uses 2 wavelengths, 43 melanin concentrations, and 11 blood oxygen saturation levels, for a combined total of 1892 simulations for diastole and systole. The simulation domain is discretized at a 10  μm resolution over a 6×6×8  mm volume. Both the source (radius = 0.2 mm) and detector (radius = 0.0667 mm) are centered at the top surface, modeled after a QR400-7-vis (OceanView, USA) reflectance probe. The probe’s concentric ring of six source fibers is approximated as a single, uniform, inward-facing disk, with the detector positioned at its center. Figure 1(a) illustrates the experimental reflectance probe configuration and its approximation in MATLAB. This approximation produces results comparable to those of the exact probe geometry, as shown in Fig. S2 in the Supplementary Material. For the scope of this study, the simulation depth can be reduced to 1.52 mm; with a concentric ring source–detector separation of 0.133 mm, only a small fraction of photons reaches the deeper layers (see Fig. S3 in the Supplementary Material). We chose our current model because it supports future exploration that requires larger source–detector separation distances, similar to those used in wearable devices.

The skin’s microstructure composition is approximated through six horizontally stacked layers: epidermis (EP), capillary loops, upper plexus, reticular dermis, deep plexus, and subcutis.^33^ Table 1 lists the structural and optical parameters of each layer, including layer thickness (d), refractive index (n), and average vessel diameter ($v_{d}$).^33^ The parameter $v_{d}$ controls the extent of self-shielding and is thus essential for aligning the simulated diffuse reflectance with experimental data presented in Sec. 3.1. Optical properties of tissues are described in terms of absorption coefficient ($\mu_{a}$), scattering coefficient ($\mu_{s}$), and anisotropy (g), which characterize how photons interact with the medium.^35^ Absorption coefficient quantifies the amount of light absorbed per distance as it travels through a medium. It is computed by summing the absorption contributions of each chromophore in the layer. A commonly used formulation, when volume fractions are known, is as follows:^35^

$$\mu_{a}=\underset{i}{\sum }f_{i}\mu_{a,i},$$ (1)

where $f_{i}$ is the volume fraction of chromophore i, and $\mu_{a,i}$ is its corresponding absorption coefficient at wavelength λ. The absorption coefficient spectra of different chromophores present in tissue from 400 to 1000 nm can be found in Fig. S4 in the Supplementary Material. The calculation of a layer’s absorption coefficient involves multiplying the volume fraction of a chromophore with its absorption coefficient and summing the absorption of all chromophores for every wavelength. Below is the equation for calculating the absorption coefficient of the epidermis^33^

$$\mu_{a,\mathrm{EP}}(\lambda )=f_{m}\mu_{a,m}(\lambda )+f_{w}\mu_{a,w}(\lambda )+(1-f_{m}-f_{w})\cdot (0.122+42.65e^{-\frac{\lambda -154}{66.2}}),$$ (2)

where $f_{m}$ and $f_{w}$ denote the melanin and water volume fractions, respectively, and the remaining term represents baseline epidermal absorption.

**Table 1:** Optical and structural parameters of skin layers.^33^^,^^34^

	$C_{m}$	$C_{b}$	$C_{w}$	$C_{f}$	d (mm)	n	$v_{d}$ (mm)
Epidermis	0.01 to 0.43	0.00	0.20	0.00	0.1	1.33	0.00
Capillary loops	0.00	0.04	0.65	0.17	0.15	1.37	0.01
Upper plexus	0.00	0.02	0.65	0.17	0.08	1.40	0.02
Reticular dermis	0.00	0.004	0.65	0.17	1.2	1.40	0.02
Deep plexus	0.00	0.04	0.65	0.17	0.5	1.40	0.04
Subcutis	0.00	0.03	0.05	0.90	5.0	1.44	0.05

Layers containing dense networks of pulsatile vessels require distinct absorption coefficient calculations for the diastolic and systolic phrases. During diastole, the blood volume fraction is lower, whereas systolic expansion increases both blood volume fraction and layer thickness. The diastolic absorption coefficient $\mu_{a}^{\left(d\right)}$ is computed using the following:^33^

$$f_{b}^{\left(d\right)}=f_{b}S[(v_{d}((1-{\mathrm{SpO}}_{2})\mu_{a,\mathrm{Hb}}+{\mathrm{SpO}}_{2}\mu_{a,{\mathrm{HbO}}_{2}}))],$$ (3)

$$\mu_{a}^{\left(d\right)}=f_{b}^{\left(d\right)}((1-{\mathrm{SpO}}_{2})\mu_{a,\mathrm{Hb}}+{\mathrm{SpO}}_{2}\mu_{a,{\mathrm{HbO}}_{2}})+\mu_{a,\text{baseline}},$$ (4)

where $f_{b}^{\left(d\right)}$ denotes the diastolic blood volume fraction, and S represents a shelf-shielding correction function that accounts for light interactions with vessel walls

$$S[\mu_{a}v_{d}]=\frac{1}{1+1.007(\frac{\mu_{a}v_{d}}{2}{)}^{1.228}}.$$ (5)

The vessel diameter determines the magnitude of self-shielding correction applied to each layer. The effect of self-shielding correction is illustrated in Fig. S5 in the Supplementary Material. Although self-shielding correction is not inherently necessary for pulse oximetry, which relies on wavelengths above 600 nm, it is essential for reproducing experimental reflectance data in the 400 to 600 nm range, where the absorption of blood is greater.

Systolic absorption coefficient $\mu_{a}^{\left(s\right)}$ is then computed by increasing the blood volume fraction and layer thickness^33^

$$f_{b}^{\left(s\right)}=f_{b}^{\left(d\right)}+pS[(v_{d}((1-{\mathrm{SpO}}_{2})\mu_{a,\mathrm{Hb}}+{\mathrm{SpO}}_{2}\mu_{a,{\mathrm{HbO}}_{2}}))],$$ (6)

$$\mu_{a}^{\left(s\right)}=E[f_{b}^{\left(d\right)}((1-{\mathrm{SpO}}_{2})\mu_{a,\mathrm{Hb}}+{\mathrm{SpO}}_{2}\mu_{a,{\mathrm{HbO}}_{2}})+\mu_{a,\text{baseline}}],$$ (7)

where p=0.5 is the pulsatile constant representing the relative increase in arterial blood volume within pulsation layers, and E≈0.79 is a layer expansion factor accounting for the increase in layer thickness during systole. Additional details about the layer expansion factor can be found in Moço.^33^ The absorption coefficient for each layer is computed with a minimum melanin volume fraction of 0.01 and presented in Figs. 1(c) and 1(d).

The scattering coefficient encompasses the combined effects of Rayleigh and Mie scattering in tissue. It is approximated by a power–law relationship that captures the wavelength-dependent nature of scattering.^33^^–^^35^ The corresponding anisotropy describes the average deflection angle of a scattered photon. Wavelength-dependent anisotropy was implemented based on trends reported in the literature.^35^ Figures 1(e) and 1(f) show the scattering coefficient and anisotropy for each layer computed with a minimum melanin volume fraction of 0.01.

## 3 Results

### 3.1 Fine-Tuning Simulation Parameters with Experimental Data

Before conducting pulsatile simulations for ratio-of-ratios calculations, the simulation parameters are fine-tuned to ensure that the model accurately reproduces experimentally measured human diffuse reflectance spectra for both light and dark skin tones in the 400 to 1000 nm range at the fingertip. Experimental reflectance spectra are obtained from OceanView HDX spectrometer with a halogen lamp, reflectance probe, and calibrated to a diffuse Spectralon standard. Measurement is at the dorsal side of the finger and participants are compared to a printed Monk Skin Tone Scale. By comparing the measured and simulated reflectance curves, several key simulation parameters, such as epidermal thickness and blood volume fraction in the capillary loops and the global scattering coefficients, are iteratively adjusted. A critical modification involves tuning the scattering coefficient’s spectral dependence (curvature changes). To implement this, two constants, $C_{1}$ and $C_{2}$, are introduced to the reduced scattering coefficient ($\mu_{s}^{\prime }$) equation. ^35^ Specifically, $C_{1}$ controls the curvature of the spectrum, whereas $C_{2}$ acts as an offset to preserve the scattering magnitude at 400 nm

$$\mu_{s}^{\prime }=\mu_{s_{0}}(\frac{577}{\lambda^{C_{1}}})\cdot C_{2}.$$ (8)

Through iterative matching of simulated reflectance to experimental data, it was determined that increasing the overall scattering magnitude and reducing the curvature improved agreement with measured reflectance for both low-melanin and high-melanin skins. Accordingly, we define

$$C_{1}=0.5,$$ (9)

$$C_{2}=400^{C_{1}-1}.$$ (10)

In addition, a melanin-dependent scattering model is introduced specifically for the epidermis to increase tissue scattering as melanin volume fraction increases; the epidermis uses a separate formulation with melanin-dependent parameters $C_{1,\mathrm{EP}}$ and $C_{2,\mathrm{EP}}$. Its reduced scattering coefficient is given by

$$\mu_{s,\mathrm{EP}}^{\prime }=\mu_{s_{0}}(\frac{577}{\lambda^{C_{1,\mathrm{EP}}}})\cdot C_{2,\mathrm{EP}},$$ (11)

where

$$C_{1,\mathrm{EP}}=\alpha (f_{m})0.5,$$ (12)

$$C_{2,\mathrm{EP}}=\beta (f_{m})400^{C_{1,\mathrm{EP}}-1}.$$ (13)

The calibration functions, $\alpha (f_{m})$ and $\beta (f_{m})$, depend on the melanin volume fraction $f_{m}$

$$\alpha (f_{m})=1+(f_{m}-0.01)\cdot \frac{22}{3},$$ (14)

$$\beta (f_{m})=1+(f_{m}-0.01)\cdot 20.$$ (15)

**Fig. 2:**
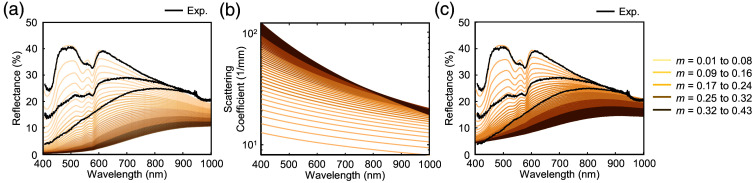
Parameter fine-tuning. (a) Comparison of simulated and experimentally measured diffuse reflectance for both light and dark skin tones without melanin-dependent scattering, following fine-tuning of the skin model to align simulated spectra with experimental measurements. The experimental reflectance spectra, ordered from top to bottom, were measured from individuals with Monk Skin Tone Types 2, 7, and 9, respectively. Reflectance simulations are performed with a melanin volume fraction ranging from 0.01 to 0.43. Under high melanin concentrations, the reflectance decreases to levels that do not align with experimental measurements. (b) Visualization of melanin-dependent scattering introduced in the epidermis. Both the amplitude and slope of the scattering coefficient spectra increase with increasing melanin concentrations. (c) Simulated reflectance under the same conditions as in panel (a) but incorporating melanin-dependent scattering to counterbalance the elevated absorption at high melanin concentrations, thereby improving agreement with experimental measurements.

Here, $f_{m}=0.01$ represents the baseline melanin volume fraction in lighter skin. As $f_{m}$ increases, α and β scale the scattering curvature and magnitude in the epidermis to mirror the observed behavior in the experimental reflectance data. Through this adjustment, the simulation more closely matches the measured spectra for high-melanin skin. Figure 2(a) presents the simulated and experimental reflectance after fine-tuning parameters but without incorporating melanin-dependent scattering. Figure 2(b) illustrates the implementation of melanin-dependent scattering in the epidermis, highlighting the effect of melanin on epidermal scattering across varying melanin volume fractions. Only the epidermis scattering coefficient is shown, as scattering in all other layers is independent of melanin. Figure 2(c) demonstrates the improved agreement between the simulated output and experimental measurements once melanin-dependent scattering is included. This parameter fine-tuning step thus ensures that the Monte Carlo model can accurately capture the optical characteristics of both light and dark skin tones, laying the groundwork for subsequent pulsatile simulations and ratio-of-ratios analyses.

### 3.3 Formulation of ${\mathrm{SaO}}_{2}$–*R* Dataset Across Varying Melanin Volume Fractions

With the skin model established, fine-tuned to experimental reflection spectra, and pulsatile dynamics captured through diastolic and systolic simulations, R values are computed across varying melanin volume fractions (0.01 to 0.43) and ${\mathrm{SaO}}_{2}$ levels (0.50 to 1.00). The pulsatile component of a PPG signal is calculated using the diffuse reflectance outputs from diastole and systole, as defined by the following equation:

$$\mathrm{AC}/{\mathrm{DC}}_{\lambda }=\frac{R_{\text{diastolic}}(\lambda )-R_{\text{systolic}}(\lambda )}{R_{\text{diastolic}}(\lambda )}.$$ (16)

**Fig. 3:**
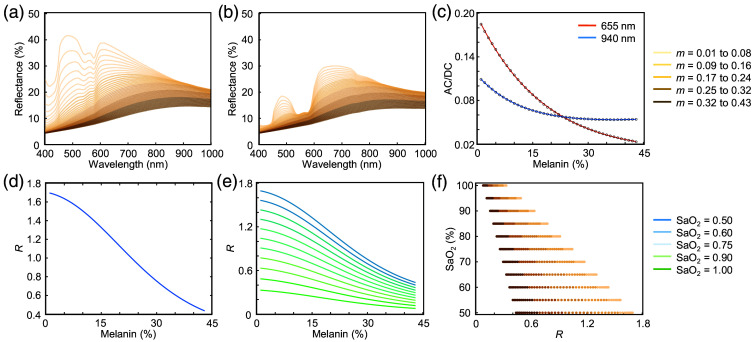
Ratio-of-ratios calculations and melanin–${\mathrm{SaO}}_{2}$ sweep. (a) Diastolic reflectance with varying melanin volume fractions at 50% ${\mathrm{SaO}}_{2}$. (b) Systolic reflectance simulated under the same conditions. The increased blood absorption is evident from 400 to 600 nm, where hemoglobin absorption is most prominent, leading to a reduction in reflectance amplitude. (c) Resulting AC/DC ratios at 655 and 940 nm with ${\mathrm{SaO}}_{2}=50\%$, calculated using Eq. (16). Exponential functions were fitted to the data at each wavelength to reduce discretization artifacts from the simulation. (d) Corresponding R values, exhibiting a non-linear decrease with increasing melanin content. (e) Applying the same methodology described in panels (a)–(d) across ${\mathrm{SaO}}_{2}$ levels ranging from 50% to 100%. (f) Mapping of R values to ${\mathrm{SaO}}_{2}$ across varying melanin volume fractions, illustrating a leftward shift in R with higher melanin content. This shift explains the overestimation of ${\mathrm{SpO}}_{2}$ in individuals with darker skin tones when the ratio-of-ratios is calibrated using data from lighter skin tones.

Figures 3(a) and 3(b) show the diastolic and systolic diffuse reflectance output at an ${\mathrm{SaO}}_{2}$ level of 50%. By subtracting systolic from diastolic reflectance and normalizing by diastolic reflectance, it isolates the blood-induced pulsatile changes in optical absorption.

The computed AC/DC values are mapped to an exponential function to reduce noise, ensuring a more continuous and physiologically realistic progression of AC/DC values with respect to melanin volume fraction [Fig. 3(c)]. Details of the curve-fitting process, including the best-fit equation and coefficients, are provided in the Supplementary Material. The R value is then calculated by taking the ratio of AC/DC components at 655 and 940 nm

$$R\approx \frac{\mathrm{AC}/{\mathrm{DC}}_{655\;\mathrm{nm}}}{\mathrm{AC}/{\mathrm{DC}}_{940\;\mathrm{nm}}}.$$ (17)

Figure 3(d) illustrates how R changes under varying melanin concentrations for a fixed ${\mathrm{SaO}}_{2}$, providing insight into the impact of melanin on the ratio-of-ratios. In clinical practice, R is extracted from PPG and mapped to ${\mathrm{SaO}}_{2}$ using a linear or quadratic function as shown below, and regression algorithms are used to calculate the coefficients^15^

$${\mathrm{SpO}}_{2}=\alpha +\beta R,$$ (18)

$${\mathrm{SpO}}_{2}=\alpha R^{2}+\beta R+c.$$ (19)

The testing procedure for pulse oximeters requires the simultaneous collection of invasive arterial blood oxygen saturation (${\mathrm{SaO}}_{2}$), and the device reported non-invasive ${\mathrm{SpO}}_{2}$. Accuracy is assessed by comparing ${\mathrm{SpO}}_{2}$ with the gold standard ${\mathrm{SaO}}_{2}$. The 2025 Food and Drug Administration draft guidance recommends a cohort of 150 participants, with ${\mathrm{SaO}}_{2}$ reference values ranging from 70% to 100%.^36^ It further recommends that representation of Monk Skin Tone Scale groups of low (1-4), medium (5-7), and high (8-10) each constitute at least 25% of the study population.

To explore the combined influence of melanin and oxygen saturation, Monte Carlo simulations are systematically performed across melanin volume fractions ranging from 0.01 to 0.43 and ${\mathrm{SaO}}_{2}$ levels from 50% to 100%. Figures 3(e) and 3(f) present the resulting R distributions. These plots clearly demonstrate how both increasing melanin and decreasing ${\mathrm{SaO}}_{2}$ influence the ratio-of-ratios. This ${\mathrm{SaO}}_{2}$–R dataset forms the foundation for developing and validating skin tone–corrected ${\mathrm{SpO}}_{2}$ models in subsequent analysis.

## 4 Discussion

### 4.1 Incorporating Melanin Correction Factor to Reduce Skin Tone Bias

**Fig. 4:**
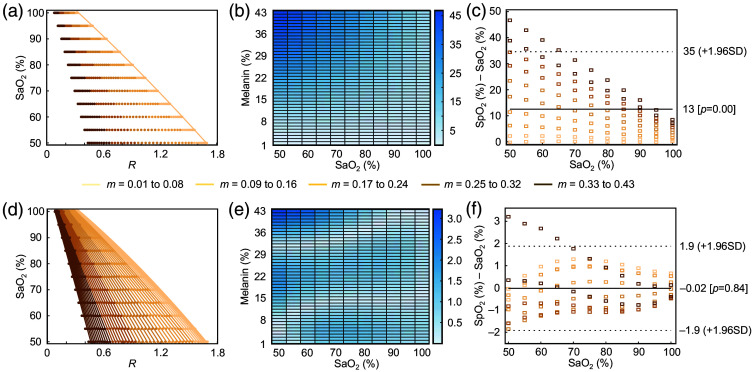
Quantifying and addressing skin tone bias. (a) Curve-fitting of Eq. (19), the quadratic ratio-of-ratios model, to simulated ${\mathrm{SaO}}_{2}$–R data at minimum melanin volume fraction. (b) Heatmap of absolute percent error across varying melanin and ${\mathrm{SaO}}_{2}$ values, defined as the absolute difference between the input ${\mathrm{SaO}}_{2}$ used in simulation and the corresponding output ${\mathrm{SpO}}_{2}$ predicted by the calibrated ratio-of-ratios model. (c) Bland–Altman plot revealing increased inaccuracies attributed to melanin. The ground truth ${\mathrm{SaO}}_{2}$ corresponds to the blood oxygenation level specified in the skin model. (d) Multiple curve-fitting performed using Eq. (20), enabled by the introduction of a new melanin-dependent variable. (e) Heatmap illustrating improved model performance, with the maximum absolute percent error reduced to 3.21%. (f) Bland–Altman plot demonstrating the elimination of skin tone bias in the improved model.

With the ${\mathrm{SaO}}_{2}$–R dataset generated across a range of melanin volume fractions, the conventional ratio-of-ratios method can now be applied to quantify skin tone induced bias. First, calibration coefficients are derived from curve-fitting the minimum melanin volume fraction ($f_{m}=0.01$), reflecting how inappropriate sampling in human pulse oximetry testing can over-rely on measurements from lighter skin tones. As shown in Fig. 4(a), the fitted quadratic equation yields a specific set of ratio-of-ratios coefficients, and Fig. 4(b) presents the resulting error map. Notably, the error increases as melanin volume fraction increases, which is consistent with the overestimation of ${\mathrm{SpO}}_{2}$ reported in the literature.^5^^–^^8^ The increased error at higher ${\mathrm{SpO}}_{2}$ levels is particularly concerning, as it implies that during critical conditions, where accurate oxygen assessment is most vital, the overestimation of ${\mathrm{SpO}}_{2}$ becomes more prominent.

A more detailed examination of the simulated ${\mathrm{SaO}}_{2}$–R dataset reveals a melanin induced shift in R values as the melanin volume fraction increases. To address this shift, it is necessary to characterize the nature of the shift. R is plotted as a function of melanin volume fraction in Fig. 3(d), revealing a non-linear relationship. This non-linearity motivates the incorporation of a quadratic melanin correction factor into ratio-of-ratio to account for the observed shift

$${\mathrm{SpO}}_{2}=aR^{2}+(b+c_{1}m^{2})R+c,$$ (20)

where m denotes the melanin volume fraction, and a, b, c, and $c_{1}$ are curve-fitting coefficients with the following values: a=−0.0462, b=−0.2795, $c_{1}=-5.4755$, and c=1.1026.

The addition of melanin-dependent variables enables multiple-curve fitting, as shown in Fig. 4(d). Compared with the results from conventional ratio-of-ratios, the proposed ${\mathrm{SpO}}_{2}$ correction model exhibits substantially improved performance. For instance, the maximum absolute percent error is reduced from 46.67% to 3.21%. Moreover, the previously observed trend of increasing error as melanin volume fraction increases is no longer evident. The increase in error at lower ${\mathrm{SpO}}_{2}$ levels has also been eliminated, demonstrating that the proposed model is more robust and resilient across variations in both skin tone and oxygen saturation. Figure 4(f) further corroborates this improvement through a Bland–Altman analysis, highlighting the model’s potential for providing more equitable and accurate ${\mathrm{SpO}}_{2}$ measurements.

### 4.2 Evaluating Candidate Blood Oxygen Saturation Correction Models

A series of candidate ${\mathrm{SpO}}_{2}$ correction models are proposed and evaluated to mitigate melanin induced bias, each incorporating distinct placements of the melanin-dependent term, as well as incorporating multiple melanin-dependent terms to further enhance the model’s ability to capture non-linearity. Table 2 presents a list of proposed ${\mathrm{SpO}}_{2}$ correction models, along with their corresponding maximum percent error obtained from curve-fitting to the simulated ${\mathrm{SaO}}_{2}$–R dataset.

**Table 2:** Evaluation of candidate ${\mathrm{SpO}}_{2}$ correction models.

N	${\mathrm{SpO}}_{2}$ correction models	Avg (%) error	Max (%) error
12	${\mathrm{SpO}}_{2}=(a+c_{1}m^{3}+c_{2}m^{2}+c_{3}m)R^{2}+(b+c_{4}m^{3}+c_{5}m^{2}+c_{6}m)R+(c+c_{7}m^{3}+c_{8}m^{2}+c_{9}m)$	0.1198	0.6457
9	${\mathrm{SpO}}_{2}=(a+c_{1}m^{3}+c_{2}m^{2}+c_{3}m)R^{2}+(b+c_{4}m^{3}+c_{5}m^{2}+c_{6}m)R+c$	0.1207	0.6195
	${\mathrm{SpO}}_{2}=(a+c_{1}m^{3}+c_{2}m^{2}+c_{3}m)R^{2}+bR+(c+c_{4}m^{3}+c_{5}m^{2}+c_{6}m)$	0.8814	4.8669
	${\mathrm{SpO}}_{2}=aR^{2}+(b+c_{1}m^{3}+c_{2}m^{2}+c_{3}m)R+(c+c_{4}m^{3}+c_{5}m^{2}+c_{6}m)$	0.1529	0.6977
	${\mathrm{SpO}}_{2}=\alpha (R-h+c_{1}m^{3}+c_{2}m^{2}+c_{3}m{)}^{2}+(k+c_{4}m^{3}+c_{5}m^{2}+c_{6}m)$	1.6311	6.8124
6	${\mathrm{SpO}}_{2}=(a+c_{1}m^{3}+c_{2}m^{2}+c_{3}m)R^{2}+bR+c$	1.9075	5.6218
	${\mathrm{SpO}}_{2}=aR^{2}+(b+c_{1}m^{3}+c_{2}m^{2}+c_{3}m)R+c$	0.1734	0.5476
	${\mathrm{SpO}}_{2}=aR^{2}+bR+(c+c_{1}m^{3}+c_{2}m^{2}+c_{3}m)$	3.2536	11.455
	${\mathrm{SpO}}_{2}=(a+c_{1}m^{3}+c_{2}m^{2}+c_{3}m)R^{2}+(b+c_{1}m^{3}+c_{2}m^{2}+c_{3}m)R+(c+c_{1}m^{3}+c_{2}m^{2}+c_{3}m)$	1.8503	9.2340
	${\mathrm{SpO}}_{2}=\alpha (R-h+c_{1}m^{3}+c_{2}m^{2}+c_{3}m{)}^{2}+k$	4.4388	17.387
4(C)	${\mathrm{SpO}}_{2}=(a+c_{1}m^{3})R^{2}+bR+c$	2.1559	7.2934
	${\mathrm{SpO}}_{2}=aR^{2}+(b+c_{1}m^{3})R+c$	1.5949	5.1174
	${\mathrm{SpO}}_{2}=aR^{2}+bR+(c+c_{1}m^{3})$	4.8003	17.582
	${\mathrm{SpO}}_{2}=(a+c_{1}m^{3})R^{2}+(b+c_{1}m^{3})R+(c+c_{1}m^{3})$	3.1700	12.249
	${\mathrm{SpO}}_{2}=\alpha (R-h+c_{1}m^{3}{)}^{2}+k$	5.6917	22.301
4(Q)	${\mathrm{SpO}}_{2}=(a+c_{1}m^{2})R^{2}+bR+c$	4.1134	15.169
	${\mathrm{SpO}}_{2}=aR^{2}+(b+c_{1}m^{2})R+c$	0.7714	3.2075
	${\mathrm{SpO}}_{2}=aR^{2}+bR+(c+c_{1}m^{2})$	3.7409	14.743
	${\mathrm{SpO}}_{2}=(a+c_{1}m^{2})R^{2}+(b+c_{1}m^{2})R+(c+c_{1}m^{2})$	1.9320	10.185
	${\mathrm{SpO}}_{2}=\alpha (R-h+c_{1}m^{2}{)}^{2}+k$	4.9996	20.079
4(L)	${\mathrm{SpO}}_{2}=(a+c_{1}m)R^{2}+bR+c$	6.8507	26.144
	${\mathrm{SpO}}_{2}=aR^{2}+(b+c_{1}m)R+c$	4.0023	15.840
	${\mathrm{SpO}}_{2}=aR^{2}+bR+(c+c_{1}m)$	3.4206	14.325
	${\mathrm{SpO}}_{2}=(a+c_{1}m)R^{2}+(b+c_{1}m)R+(c+c_{1}m)$	3.8059	15.139
	$S{\mathrm{pO}}_{2}=\alpha (R-h+c_{1}m{)}^{2}+k$	4.4276	17.921

Models are grouped by N, the number of coefficients. The bracketed labels (C, Q, and L) indicate cubic, quadratic, and linear melanin-dependent terms, respectively.

As expected, the average and maximum absolute percent errors decrease with an increasing number of coefficients, which is consistent with the principle that higher degrees of freedom in curve-fitting yield lower fitting error. However, model complexity must be balanced against the risk of overfitting, which can reduce generalizability. In pursuit of this balance, we propose the following expression, which introduces only a single quadratic melanin-dependent term

$${\mathrm{SpO}}_{2}=aR^{2}+(b+c_{1}m^{2})R+c,$$ (21)

where m denotes the melanin volume fraction. This model outperforms the standard ratio-of-ratios approach while achieving average and maximum percent errors comparable to those of more complex expressions. Moreover, the melanin volume fraction can be estimated from various established indices, such as ITA, VLS, or Monk Skin Tone Scale, offering practical applicability for clinical implementation. Provided the melanin index is accurately and consistently measured, the expression is expected to produce skin tone–invariant pulse oximetry estimates. We present a sensitivity analysis in the Supplementary Material to demonstrate how inaccuracies in melanin estimation propagate to errors in ${\mathrm{SpO}}_{2}$ estimation. The analysis indicates that estimates of melanin volume fraction must achieve an accuracy within 5%. This enables researchers to determine whether their chosen skin tone color space provides sufficient precision for integration with our proposed skin tone–corrected pulse oximetry model.

## 5 Conclusion

This work presents a practical strategy for correcting skin tone induced bias in reflective pulse oximetry by incorporating a melanin-dependent correction factor into the conventional ratio-of-ratios methods. Monte Carlo simulations were used to model light–tissue interactions across a realistic, multilayer skin structure and validated through experimental diffuse reflectance measurements, sensitivity analyses, and convergence tests. By systematically implementing pulsatility and varying both melanin volume fraction and ${\mathrm{SaO}}_{2}$, we generated a comprehensive dataset of R values and thereby quantified the extent of skin tone bias. The proposed melanin-dependent correction term significantly reduces measurement error due to both skin pigmentations across oxygen saturation, offering a more robust and equitable means of monitoring arterial oxygen levels.

Monte Carlo simulations offer a powerful and flexible framework for independently varying and precisely controlling melanin volume fraction and ${\mathrm{SaO}}_{2}$ levels, which is challenging to replicate *in vivo*. Although this study focuses on pulse oximetry, the underlying methodology can be extended to investigate a wide array of light–tissue interactions, including optical transmission and more complex device geometries. As remote patient monitoring becomes increasingly integral to modern healthcare, the need for bias-resistant medical devices is more crucial than ever. The findings presented here not only address existing disparities in pulse oximetry performance but also represent a step toward creating more inclusive and equitable patient care in the future.

## Supplementary Material

10.1117/1.BIOS.2.3.032507.s01

## Data Availability

All scripts and data used in the publication are available here: github.com/NickC18/Skin-Tone_Corrected_Pulse_Oximetry_Models
