# Supplementary material for: Skin tone corrected pulse oximetry models evaluated through reflective pulsatile Monte Carlo simulations
Source: Biophotonics Discov. 2025 Aug 13;2(3):032507. doi: 10.1117/1.BIOS.2.3.032507 (PMC13052488; doi:10.1117/1.BIOS.2.3.032507)
Supplement: Supplementary file 1 [file BIOS_002_032507_SD001.pdf]

Supplemental Information

Supplementary Note 1: Mitigation of Simulation Discretization Artifacts

$$f(x) = ae^{bx} \tag{S1}$$

	a	b	R^2
655nm	0.194	-5.184	0.9994
940nm	0.096	-1.842	0.8506

$$f(x) = ae^{bx} + ce^{dx} \tag{S2}$$

	a	b	c	d	R^2
655nm	0.195	-5.400	0.001	4.549	1.0000
940nm	0.076	-7.759	0.038	0.675	0.9999

(S1) Single exponential fit applied to the simulated AC/DC ratios shown in Figure 3c. **(S2)** Double exponential fit performed to maximize R^2. The double exponential model in (S2) provides a better fit than the model in (S1) and is used to generate the fitted line presented in Figure 3c.

Supplementary Note 2: Sensitivity of SpO_2 to Melanin Estimation Errors

We begin with our proposed SpO_2 correction model:

$$SpO_2 = aR^2 + (b + c_1 m^2)R + c \tag{S3}$$

The coefficients take on the following values after the curve-fitting process shown in Figure 4d:

$$a = -0.0462,\ b = -0.2795,\ c_1 = -5.4755,\ c = 1.1026$$

Next, the sensitivity of SpO_2 with respect to melanin is computed as:

$$S_{SpO_2,m} = \frac{\partial SpO_2}{\partial m} \cdot \frac{m}{SpO_2(R,m)} \tag{S4}$$

$$S_{SpO_2,m}(R,m) = \frac{2c_1 m^2 R}{SpO_2(R,m)} \quad \text{(S5)}$$

Below is a table showing the sensitivity across various melanin and SpO_2 values. The corresponding R values are obtained from Figure 3f.

m	SpO_2	R	$S_{SpO_2,m}$
0.01	1.0	0.3295	-0.0004
0.01	0.7	1.1749	-0.0018
0.01	0.5	1.6963	-0.0037
0.10	1.0	0.2818	-0.0309
0.10	0.7	1.0334	-0.1617
0.10	0.5	1.5042	-0.3294
0.20	1.0	0.2068	-0.0906
0.20	0.7	0.7703	-0.4820
0.20	0.5	1.1276	-0.9879
0.43	1.0	0.0782	-0.1583
0.43	0.7	0.2948	-0.8528
0.43	0.5	0.4348	-1.7609

The table below presents the maximum tolerable melanin error required to maintain SpO_2 error within 3% (the cutoff for FDA clinical pulse oximeter accuracy), calculated using the two highest sensitivity values highlighted above.

SpO_2 (%)	Maximum Tolerable Melanin Error (%)
50	3.4
70	5.0

Based on the SpO_2 range assessed by the FDA (SpO_2 > 70%), the maximum allowable tolerance for melanin volume fraction is 5.0%.

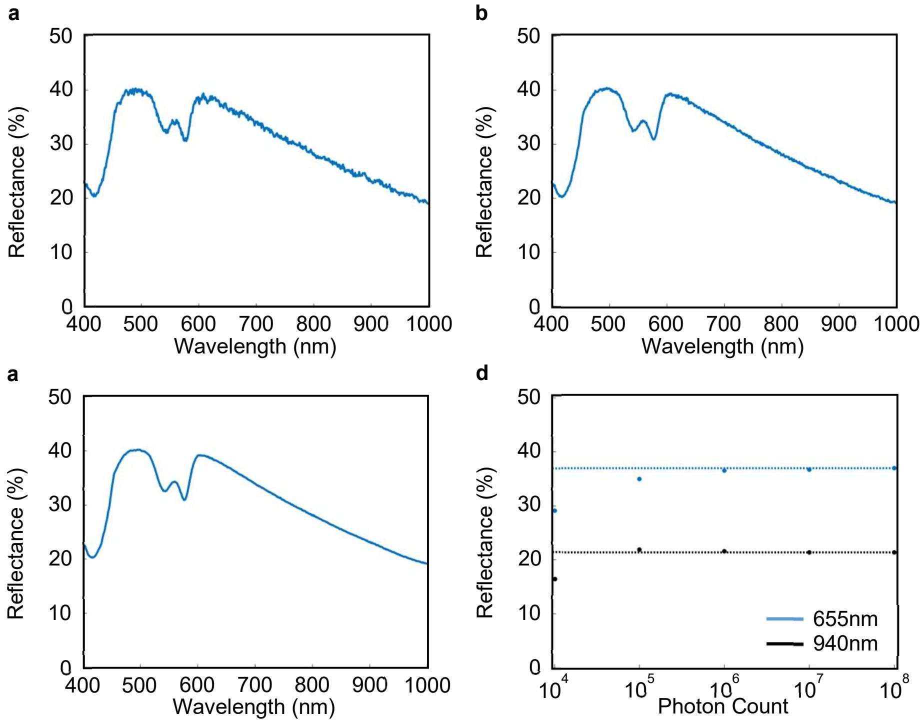

Supplemental Figure S1 | Convergence Analysis of Simulated Reflectance. (a - c) Simulated diffuse reflectance spectra using photon counts of 10^6, 10^7, 10^8, respectively. As the number of photons increases, noise in the reflectance spectra visibly decreases. **(d)** Reflectance values at 655nm and 940nm are plotted for each photon count. The horizontal line represents the reflectance value obtained with 10^8 photons. The absolute difference from this reference line decreases with higher photon counts, clearly capturing convergence. This minimal deviation at higher photon levels indicates that 10^8 photons closely approximate the theoretical true result, thus it is a suitable choice of photon count for accurate simulations.

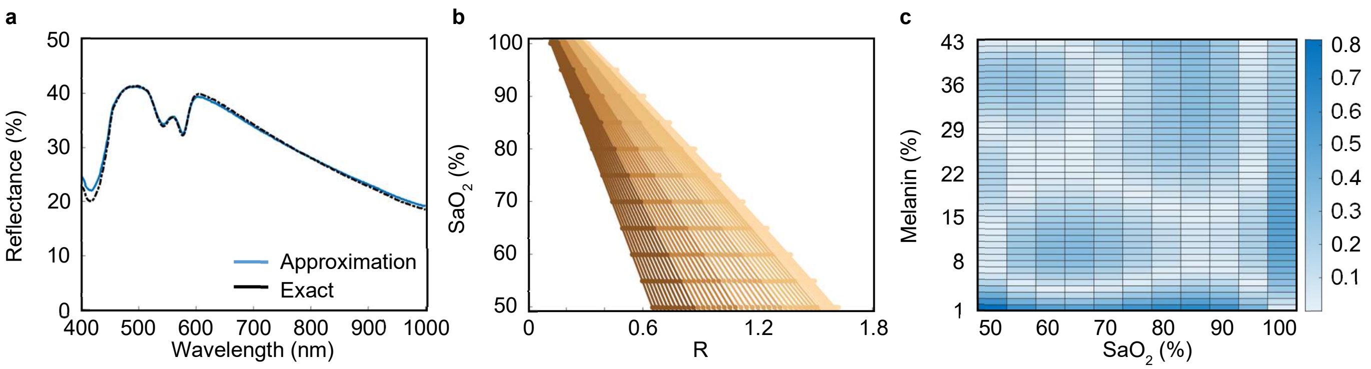

Supplemental Figure S2 | Accuracy Assessment of Reflectance Probe Approximation. (a) Simulated diffuse reflectance obtained using the approximated and exact probe geometries shows comparable values at 655 and 940 nm. The exact geometry model was fine-tuned to exhibit higher tissue scattering than the approximated geometry model. Specifically, the C_1 constant was increased from 0.5 to 0.555. **(b - c)** Multiple curve-fitting of simulated Ratio-of-Ratios values generated with the exact probe geometry, together with the corresponding heatmap. The melanin-induced shift and the extent of skin-tone bias correction closely match those produced by the approximation (Figures 4d and 4e), confirming that the simplified geometry accurately reproduces reflectance probe performance.

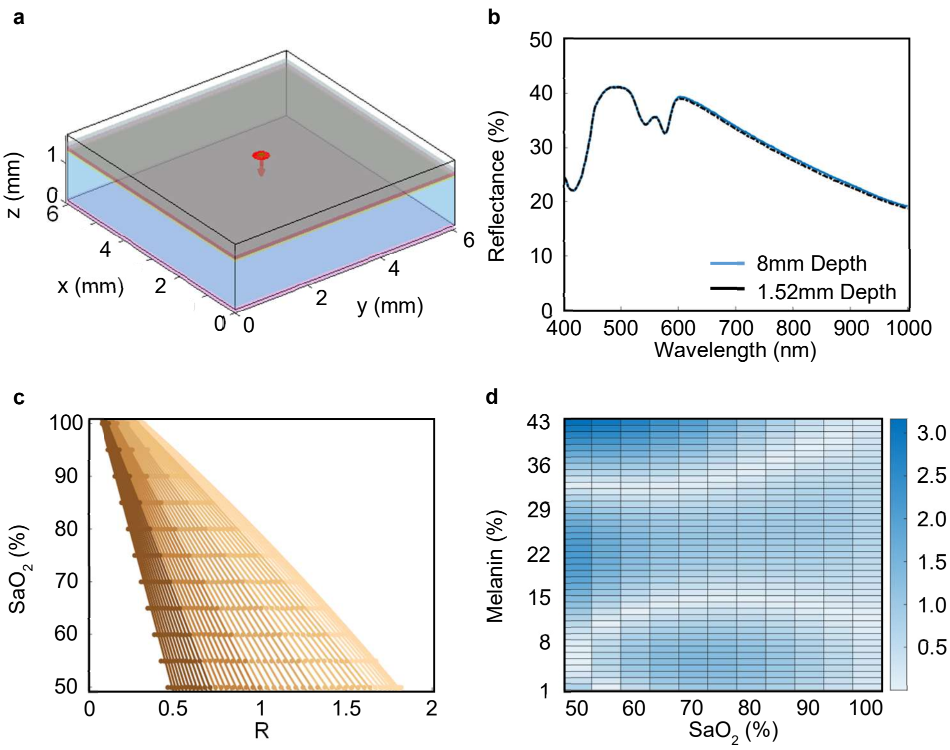

Supplemental Figure S3 | Analysis of Truncated Skin Model. (a) A simulation volume with depth reduced to 1.52 mm, consisting of only the soft tissues superficial to the distal phalanx[37]. **(b)** Simulated diffuse reflectance computed with the original 8 mm model and the truncated 1.52 mm model. **(c – d)** Multiple curve-fitting results and corresponding heatmap using the new simulation volume with a depth of 1.52 mm, following the same Ratio-of-Ratios calculations presented in the manuscript. The same SpO_2 correction model remains effective and substantially reduces skin-tone bias, confirming that reducing the volume depth has negligible impact.

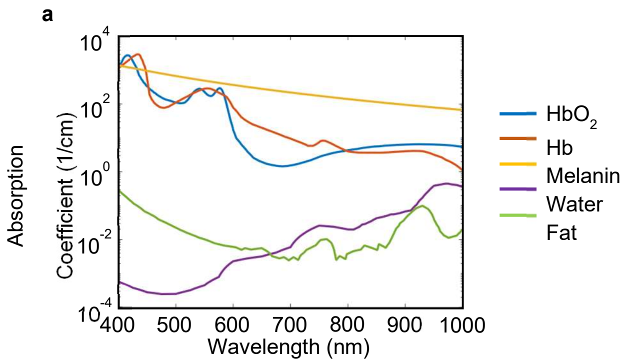

Supplemental Figure S4 | Absorption Coefficient of Skin Chromophores. (a) Absorption spectra for oxyhemoglobin, deoxyhemoglobin, melanin, and water are obtained from Jacques,[35] and fat absorption is taken from van Veen et. al.[38] The absorption coefficient of a medium is modeled as the weighted sum of individual chromophore contributions, as described in Equation (11). Although melanin exhibits stronger absorption than other chromophores at 660nm and 940nm, it is omitted in conventional Ratio-of-Ratios, contributing to skin-tone bias.

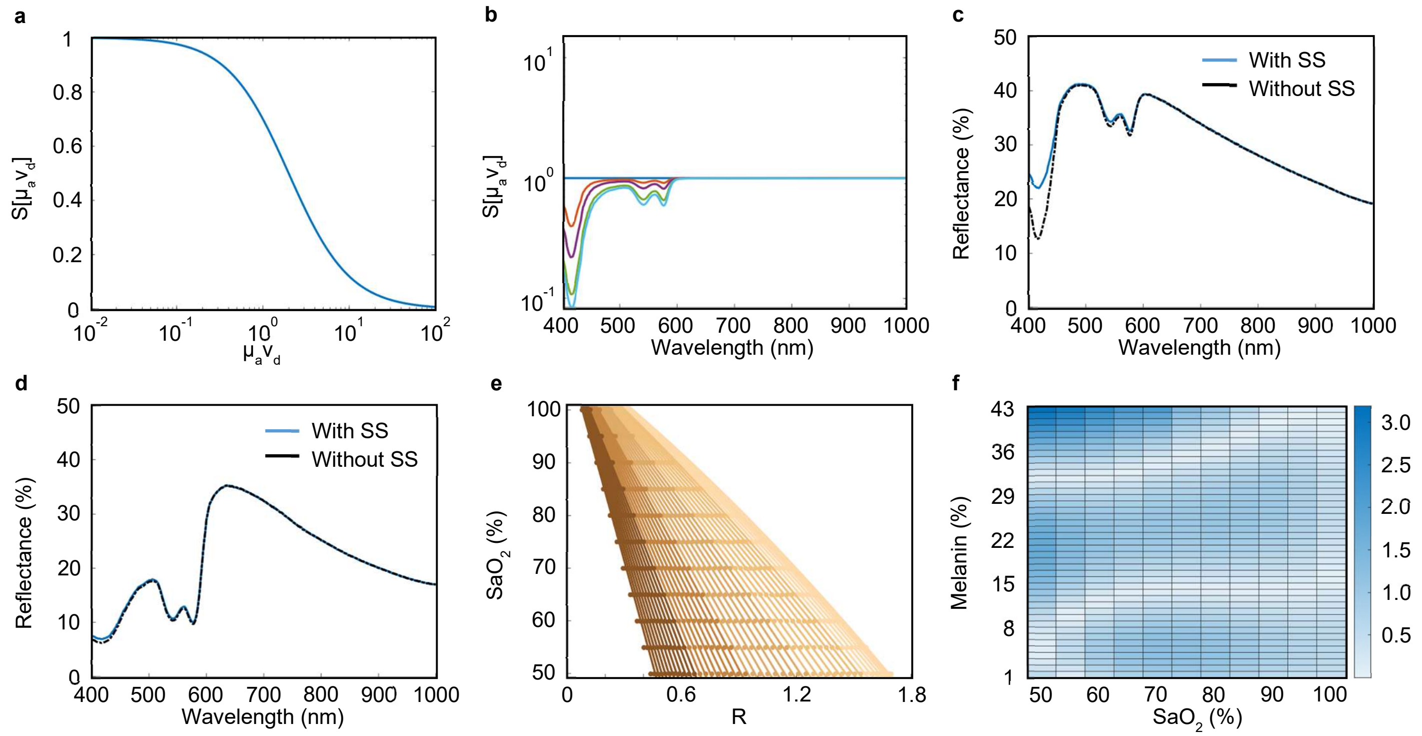

Supplemental Figure S5 | Effect of Self-Shielding Correction. (a) Self-shielding correction factor S plotted against the product of blood absorption and vessel diameter. **(b)** Layer specific S factors. A value less than one in the 400 to 600 nm region indicates a reduction in blood absorption due to self-shielding correction. **(c - d)** Simulated diffuse reflectance with and without self-shielding correction during diastole and systole respectively. **(e - f)** Multiple curve-fitting of simulated Ratio-of-Ratios values without self-shielding, along with the corresponding heatmap. The results are comparable to Figures 4d and 4e, indicating that the inclusion of self-shielding is not inherently necessary for pulse oximetry analysis.
